#  Infra-Auricular Subcutaneous Myxoma: Surgical Challenges and Histopathological Insights

**DOI:** 10.22038/ijorl.2025.80373.3704

**Published:** 2025

**Authors:** Sanjeev Yadav, Ashish Gupta

**Affiliations:** 1 *Department of ENT & Head Neck Surgery, UPUMS- Saifai, Uttar Pradesh, India.*; 2 *Department of General surgery, Dr B.R. Ambedkar Institute of Medical Sciences, Mohali, Punjab, India.*

**Keywords:** Neoplasms, Mesenchymal, Connective Tissue, Reconstructive Surgical Procedures, Fibroblasts

## Abstract

**Introduction:**

Myxomas are rare, benign mesenchymal tumors predominantly found in connective tissues, rarely occurring in the head and neck. Composed of stellate cells in a mucoid matrix, their incidence in intramuscular locations is about 1 in 1,000,000, with fewer than 200 cases reported since 1948. This report discusses the surgical treatment and pathology of a subcutaneous myxoma in the infraauricular region.

**Case Report:**

A middle-aged male presented with a painful, discharging polypoidal mass in the infraauricular area, initially misdiagnosed as a dermoid cyst from imaging and biopsy. Surgical excision and reconstruction using a bilobed flap were performed. Histopathological analysis confirmed myxoma. At the six-month follow-up, the patient demonstrated excellent wound healing and functional recovery, emphasizing the effectiveness of the bilobed flap in infraauricular reconstruction.

**Conclusion:**

This case highlights the diagnostic challenge of myxomas, especially in unusual locations. Effective management relies on surgical removal with histological confirmation, demonstrating the importance of considering myxomas in differential diagnoses of neck masses. The successful use of a bilobed flap for reconstruction emphasizes the necessity for appropriate surgical planning to manage aesthetic and functional outcomes.

## Introduction

Myxomas are benign, locally invasive mesenchymal neoplasms composed of undifferentiated stellate cells embedded in a mucoid stroma. They are rare and seldom occur in the neck region. Overall incidence of intramuscular myxoma is estimated to be 1 in 1,000,000 (1). Since the establishment of diagnostic criteria in 1948, fewer than 200 reported cases of myxomas in the head and neck have been reported (1). This case report highlights the clinical features, surgical management, and histopathological findings of an infraauricular subcutaneous myxoma. On imaging and initial biopsy, differentials such as dermoid cyst, epidermoid cyst, and neurofibroma were considered, which highlights the diagnostic ambiguity often encountered with infraauricular masses. The uniqueness of this case lies in its uncommon location.

## Case Report

**Fig. 1:**
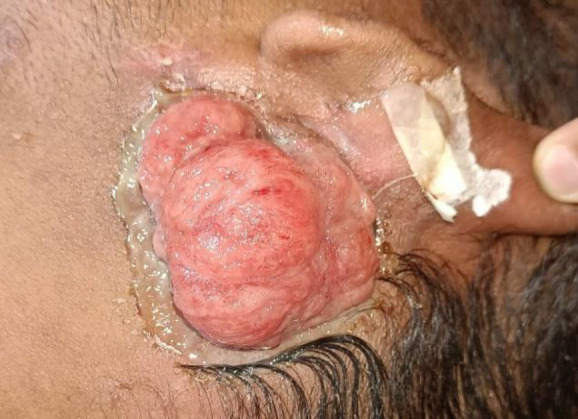
Patient observed in the outpatient department with a polypoidal mass located in the infraauricular region, alongside discharge surrounding the area

A middle-aged male patient presented with a polypoidal mass on the left side of his neck that had developed over 3-4 months. The patient complains of discharge over the swelling and occasional pain. There was no history of trauma, fever, dysphagia, hoarseness, weight loss and no other remarkable medical or family history. Clinical examination revealed a firm, non-mobile fungating mass in the infraauricular region posterior to mandibular ramus measuring 3x3 cm (Fig. 1). 

There was no other significant lymphadenopathy. Examination of oral cavity, oropharynx, nasopharynx, ear examination was normal. All cranial nerves were intact and symmetric. Contrast enhanced computed tomography of neck showed 4 cm mass at the level C5-C6, lateral to trapezius in subcutaneous plane. A biopsy suggested dermoid cyst with secondary inflammation. Patient then subsequently underwent wide local excision with reconstruction using bilobed flap. The mass was located superficial to the parotid fascia, and no deep lobe extension was noted. The superficial parotid tissue adjacent to the tumor was preserved. Facial nerve dissection was not required as the mass did not encroach upon its anatomical course. However, meticulous identification of nerve landmarks was performed intraoperatively to avoid iatrogenic injury. After local excision there was a defect of 5x5 cm with depth up to muscular plane. A bilobed flap marked with dimensions as shown in figure and advanced to cover the defect (Fig. 2 & 3). 

**Fig. 2:**
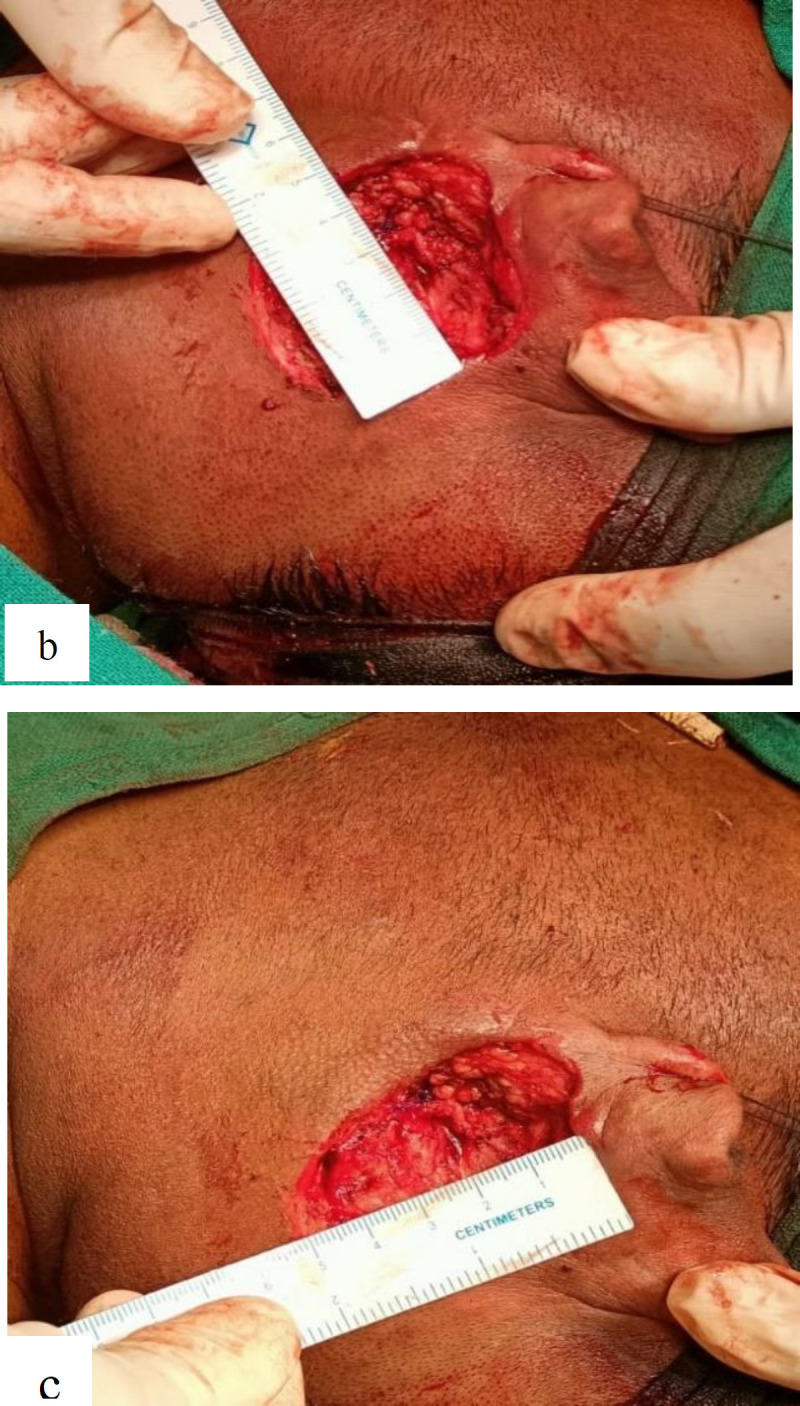
Illustrating surgical sequence. 2(a): Surgical precision: excising 3x3 cm mass with 1 cm margins.

**Fig.2(b,c):**
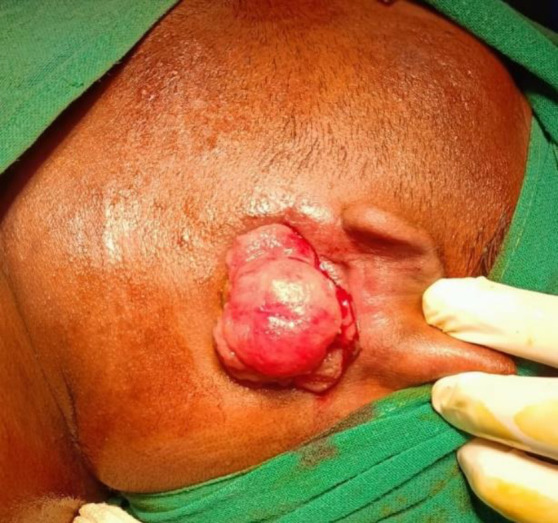
Reveiling the post excisional void measuring 5x5cm

**Fig.2(d):**
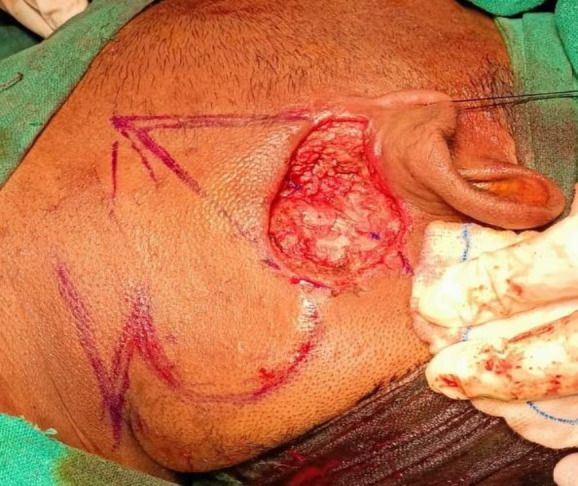
Architecting tissue reconstruction, unveiling the bilobed flap strategy.

**Fig. 3:**
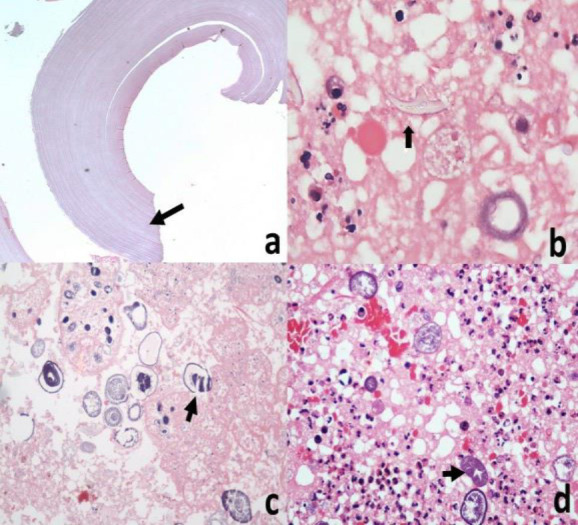
Geometric blueprint if bilobed advancement flap. The radius(r) of defect is 2.5 cm with points AB=BC=CD. The outer circle (OC) has a radius of r1=7.5cm. The inner circle (IC) has a radius of r2=5cm. This design of a bilobed flap with Burrow’s triangle minimizes tissue protrusion.

**Fig. 4:**
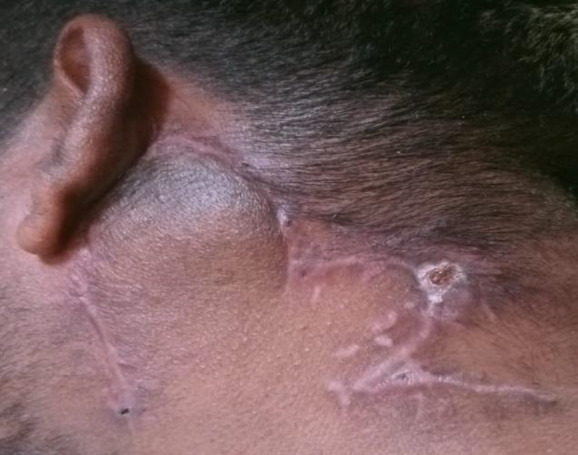
Monitoring progress: wound healing status at 3 months postoperative

Immediate post operatively a 12 French suction drain was kept in situ that was removed on post operative day 1. Post operative picture after 6 months shows well healed wound (Fig. 4).

*Histopathological Findings:* On gross examination, the specimen measured 4.2 × 3.2 × 1.5 cm and appeared polypoidal and ulcerated, with an underlying smooth skin surface. Cut sections revealed grey-white to grey-brown areas with a gelatinous consistency. Microscopically, the tumor was well-circumscribed and surrounded by a fibrous pseudocapsule, exhibiting an expansive growth pattern within a copious myxoid matrix. The tumor cells were predominantly spindle-shaped, with occasional stellate cells, showing bland nuclear chromatin, inconspicuous nucleoli, and poorly defined cytoplasm. These were arranged in short fascicles and whorls within the myxoid background (Fig. 5).

**Fig. 5:**
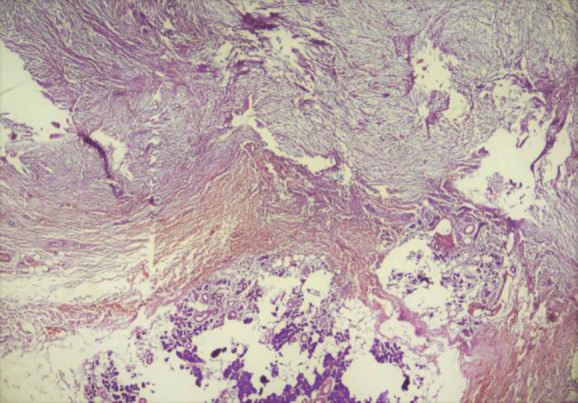
Histopathological insights. Fig 5(a) Hematoxylin-Eosin stain; 4x, low power view shows circumscribed myxoid tumor on top and normal looking salivary glamd lobules(parotid) at bottom separated by a pseudocapsule formed of compressed connective tissue

**Fig. 5(b):**
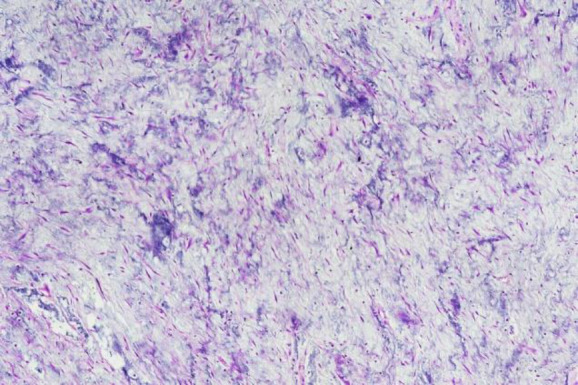
Hematoxylin-Eosin stain; 10x, Hypocellular lesion composed of spindle shaped cells and occasional stellate cells in abundant myxoid stroma

**Fig. 5 (c):**
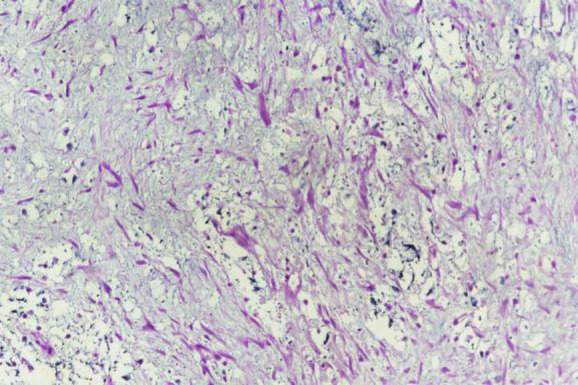
Hematoxylin-Eosin stain; 20x, Cells are spindle shaped with moderate amount of eosinophilic cytoplasm. Interspersed stellate cells (A) are also seen. Background shows abundant myxoid stroma

**Fig. 5 (d):**
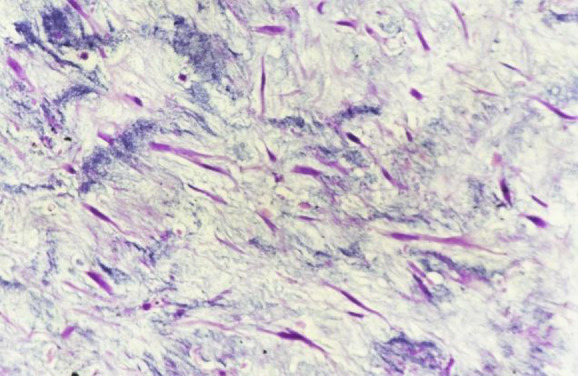
Hematoxyline-Eosin stain; 40x, on high power, cells show oval to elongated bland nuclei and inconspicuous nucleoli. No mitosis or nuclear pleomorphism observed.

No mitotic activity or nuclear pleomorphism was noted. Adjacent salivary gland lobules were present at the periphery but were not infiltrated or involved by the tumor. Histologically, the lesion was most consistent with a soft tissue myxoma; however, other myxoid neoplasms were considered. Myxoid neurofibromas typically show wavy nuclei and S100 positivity, while nerve sheath myxomas often demonstrate Antoni A and B areas or Verocay bodies — none of which were evident on light microscopy. The absence of mitosis, cellular atypia, and any biphasic architecture further supported a benign myxoid tumor consistent with a myxoma. While immunohistochemistry (e.g., S100, EMA, CD34) would have aided definitive classification, resource limitations precluded its use in this case. This remains a limitation of our report, although morphological findings were sufficiently characteristic for diagnosis.

## Discussion

Myxomas, originating from fibroblastic primitive mesenchyme, are benign tumors producing excess mucopolysaccharide and lacking mature collagen production (2). The uncertain aetiology of myxomas is widely discussed. Researchers propose derivation from primitive embryonic mesenchyme or fibroblasts capable of abundant mucopolysaccharide elaboration (2). These tumors can manifest in various body locations, such as bones, heart, skin, genitourinary tract, retroperitoneal tissues, intestinal tract, pharynx, joints, and skeletal muscles. In the head and neck, these myxomas are rare, with only few cases reported in the literature to date. Myxomas exhibit indolent growth, and patients typically present with a painless mass, although up to 20% may experience pain or tenderness (3). Soft tissue myxomas, originating from widely distributed mesenchymal tissue and may lack a complete fibrous capsule. Intraoperatively, these masses appear greyish and gelatinous, with varying consistency (4). Soft tissue myxomas exhibit local infiltration and expansion but do not metastasize. Microscopic examination involves identifying stellate cells and a reticular fibre meshwork in a mucoid matrix containing hyaluronic acid. Differentiated elements like chondroblasts, rhabdomyoblasts, or lipoblasts indicate myxoid degeneration, excluding a myxoma diagnosis (4). Treatment for head and neck myxomas involves surgical excision with adequate margins. The optimal surgical approach is debated, ranging from enucleation or curettage to extensive surgery with wide margins. Due to their recurrent nature, thorough removal with clear margins is crucial. The question of extensive resection remains debatable, especially for lesions near vital structures, where a conservative approach with close follow-up may be warranted. The decision to utilize a bilobed flap was guided by the location, shape, and size of the post-excisional defect (approximately 5×5 cm) in the infra-auricular region. Alternative techniques such as rhomboid or rotation flaps were considered; however, these may lead to increased tension or distortion of adjacent anatomical structures, especially near the mandibular border.

The bilobed flap, originally popularized by Esser and refined by Zitelli, offers geometric flexibility in closing circular or oval defects while minimizing dog-ear formation and preserving contour (5). Its design allows the recruitment of adjacent lax skin in a controlled vector, which is particularly advantageous in the upper neck where mobility and cosmesis are both concerns. In our case, the bilobed design ensured tension-free closure with minimal distortion and an acceptable scar orientation. Our adaptation followed principles described by McGregor and Soutar, modifying the lobe orientation and introducing Burrow’s triangles to further reduce tissue redundancy (6). This approach yielded excellent functional and aesthetic outcomes at 6 months follow-up. Recent literature supports the bilobed flap as a reliable reconstructive option for small-to-moderate sized facial and postauricular defects with superior cosmetic results (1,4).

## Conclusion

This case report highlights the rare occurrence of subcutaneous myxoma in the infraauricular region. Surgical excision with reconstruction using bilobed flap was successful. A thorough histopathological examination revealed the diagnosis of myxoma. The literature review underscores the infrequency of myxomas in the neck region and the importance of accurate diagnosis and management.

### Author contributions

 All authors contributed to the study conception and design. Material preparation, data collection and analysis were performed by SY, AS and TK. The first draft of the manuscript was written by SY and all authors commented on previous versions of the manuscript. All authors read and approved the final manuscript.
